# On-call abdominal ultrasonography: the rate of negative examinations and incidentalomas in a European tertiary care center

**DOI:** 10.1007/s00261-022-03525-1

**Published:** 2022-04-29

**Authors:** Tim E. Sluijter, Derya Yakar, Thomas C. Kwee

**Affiliations:** Medical Imaging Center, Department of Radiology, Nuclear Medicine and Molecular Imaging, University of Groningen, University Medical Center Groningen, Hanzeplein 1, P.O. Box 30.001, 9700 RB Groningen, The Netherlands

**Keywords:** Abdomen, Acute, Ultrasonography, Incidental findings, Negative results, Night shift work

## Abstract

**Objectives:**

To determine the proportions of abdominal US examinations during on-call hours that are negative and that contain an incidentaloma, and to explore temporal changes and determinants.

**Methods:**

This study included 1615 US examinations that were done during on-call hours at a tertiary care center between 2005 and 2017.

**Results:**

The total proportion of negative US examinations was 49.2% (795/1615). The total proportion of US examinations with an incidentaloma was 8.0% (130/1615). There were no significant temporal changes in either one of these proportions. The likelihood of a negative US examination was significantly higher when requested by anesthesiology [odds ratio (OR) 2.609, *P* = 0.011], or when the indication for US was focused on gallbladder and biliary ducts (OR 1.556, *P* = 0.007), transplant (OR 2.371, *P* = 0.005), trauma (OR 3.274, *P* < 0.001), or urolithiasis/postrenal obstruction (OR 3.366, *P* < 0.001). In contrast, US examinations were significantly less likely to be negative when requested by urology (OR 0.423, *P* = 0.014), or when the indication for US was acute oncology (OR 0.207, *P* = 0.045) or appendicitis (OR 0.260, *P* < 0.001). The likelihood of an incidentaloma on US was significantly higher in older patients (OR 1.020 per year of age increase, *P* < 0.001) or when the liver was evaluated with US (OR 3.522, *P* < 0.001).

**Discussion:**

Nearly 50% of abdominal US examinations during on-call hours are negative, and 8% reveal an incidentaloma. Requesting specialty and indication for US affect the likelihood of a negative examination, and higher patient age and liver evaluations increase the chance of detecting an incidentaloma in this setting. These data may potentially be used to improve clinical reasoning and restrain overutilization of imaging.

**Supplementary Information:**

The online version contains supplementary material available at 10.1007/s00261-022-03525-1.

## Introduction

Medical imaging utilization has rapidly grown in the developed world over the past decades [1]. A part of this increased imaging utilization has been caused by justly use of more imaging, resulting in benefits for patients like improved quality of life [2]. However, another part of this growth is due to overuse of imaging, leading to a host of potential problems, of which incidentalomas with associated healthcare costs is a notable one [2, 3]. This overuse has been attributed to different causes, including financial incentives, defensive medicine, and patient expectations [2].

As a result of the growth in imaging utilization, the workload per individual radiologist has increased considerably over the past decades, and this trend is expected to continue [4, 5]. Previous research in a large general hospital in Western Europe has shown that the overall workload during on-call hours has quadrupled in the past 15 years, particularly due to the growth in the number of CT studies (brain CT, remotely followed by body CT) [4]. Abdominal ultrasonography (US) examinations are frequently requested during on-call hours, and they put a burden on a radiologist’s workload. They also interrupt the interpretation of other acute imaging examinations (such as CT, MRI, and radiographs) during on-call hours, and US examinations are relatively time-consuming in hospitals where a radiologist rather than a sonographer performs the study. Transfer of the US machine to the patient (or vice versa) also requires valuable time. Work overload and interruptions may have detrimental effects on the quality and safety of radiological patient care [6–9]. Note that the scenario concerning the use of US during on-call hours applies to the Netherlands and several other European countries. However, the use of imaging in this setting is somewhat different in the USA, where emergency room centers serve as triage center for those without primary care (with imaging as perhaps the fastest triaging method), it is hard for emergency physicians to get a specialist consultation without imaging already performed, US is primarily performed by sonographers (who do all examinations independently, but with available oversight), and CT is more frequently used as initial imaging modality in acute abdominal pathology (except for right upper quadrant pain, exclusion of postrenal obstruction, and superficially located organs) [10, 11]. Judicious use of abdominal US during on-call hours is crucial to prevent harming patients due to incidental findings and overburdening of the radiology workforce. Overutilization of imaging may be reflected by a too high percentage of examinations with negative findings. However, it is currently unclear what can be considered a too high percentage because it is unknown how many abdominal US examinations during on-call hours are negative. The frequency of negative US examinations in this setting may be a useful benchmark for interinstitutional comparison, for temporal monitoring, and for providing feedback to requesting clinicians. Since US does not subject patients to radiation exposure, healthcare providers have one less drawback to think about when requesting this type of imaging. Therefore, it is hypothesized that the frequency of negative abdominal US examinations during on-call hours is substantial and has increased over the past years. The frequency of incidentalomas on US in this setting is also expected to be considerable, but without an a priori reason to assume that this percentage has notably changed over time. The purpose of this study was therefore to determine the proportions of abdominal US examinations during on-call hours that are negative and that contain an incidentaloma, and to explore temporal changes and determinants.

## Materials and methods

### Study design and subjects

The ethical review board of the University Medical Center Groningen approved this study, and the requirement for informed consent was waived. This study is a retrospective cohort study, performed in a tertiary care institution in The Netherlands with more than 2 million people in its direct catchment area.

Patients who underwent abdominal US performed by the radiology department during on-call hours (i.e., between 17.00 p.m. and 8.00 a.m. on weekdays, in weekends, or on official holidays) between January 2005 and November 2017 were considered for inclusion in this study. All of these US examinations during on-call hours were performed by radiology residents. The organs that were evaluated with US depended on the clinical request and the judgment of the resident based on clinical and live US findings. Residents either independently performed and reported the US examination, or were assisted and supervised by an on-call radiologist when requested by the resident. Note that a radiology residency takes 5 years in the Netherlands, that there is year-round influx of new residents, and that residents are allowed to perform on-call duties after completing the first year of residency and obtaining a sufficient “entrustable professional activity” score for US in this setting.

Using the Random Calendar Date Generator [12], 87 unique calendar dates were randomly selected between January and November. Out of all abdominal US examinations performed during on-call hours on these 87 unique calendar days in each of the years from 2005 to 2017, 250 cases per year were randomly selected. December of all 13 years was dismissed since a change in patient file software did not allow for the extraction of cases from December 2017. This random selection process yielded 3250 cases that were potentially eligible for inclusion. Exclusion criteria were as follows: US not involving the abdomen, routine protocolized US (including routine Focused Assessment with Sonography for Trauma [FAST], intra- and postoperative liver transplant US, postoperative kidney transplant US, and pre-transplant donor assessment), duplicate records of the same US examination, non-clinical US examinations (e.g., US for education or research purposes), lack of an imaging request or imaging report in the electronic patient file, and US-guided diagnostic or therapeutic interventions.

### Data collection

Patient files of all 3250 cases, including US reports, were reviewed by a research fellow (T.E.S.). The following parameters were extracted for each included case: patient age and gender, specialty that requested the US examination, and indication for US (categorized as abdominal aorta aneurysm, acute bowel pathology, acute liver failure, acute oncology, appendicitis, gallbladder and biliary ducts, infection/inflammation, mixed, other, transplant [outside of routine protocols], trauma [non-FAST] and urolithiasis/postrenal obstruction.

Each US examination was classified as either positive (i.e., findings that were related to the reason the US examination was made) or negative (i.e., the absence of findings related to the reason that the US examination was made and no disease deterioration or other new and clinically relevant findings compared to a previous imaging examination if available) [13]. If an US examination could not be clearly classified as positive or negative, it was classified as indeterminate.

For each US examination it was also recorded whether it revealed an incidentaloma or not. Incidentalomas were defined as incidental US findings serendipitously diagnosed in an asymptomatic patient or symptomatic patient undergoing imaging for an unrelated reason [3]. Both incidental findings that may potentially cause morbidity or death when left untreated, and incidental findings for which it is unknown if they require treatment, were considered incidentalomas, in line with previous literature [14]. Clearly benign findings such as simple liver or renal cysts were not considered incidentalomas [14]. Incidentalomas that had already been detected on previous imaging examinations were not counted either.

### Data analysis

The proportions of US examinations that were negative and those that yielded at least one incidentaloma were calculated, for all years together and for each year between 2005 and 2017. Temporal changes in aforementioned proportions between 2005 and 2017 were analyzed using the Mann–Kendall test. Logistic regression analyses were performed to determine the association between a negative US examination with the following variables: patient age, patient gender, specialty that requested the US examination, and indication for US. Logistic regression analyses were also performed to determine the association between the detection of an incidentaloma on US (i.e., at least one incidentaloma on a patient level) with the following variables: patient age, patient gender, and organ(s) evaluated with US. Variables that were significant on univariate analysis were subjected to multivariate analysis. Categories with the highest number of observations were used as reference for nominal variables. Categories with less than 10 counts were excluded from logistic regression analyses. Two-sided *P*-values < 0.05 were considered statistically significant. Statistical analyses were executed using R version 4.1.1. software (https://www.r-project.org) and MedCalc version 19.1.6 software (MedCalc, Mariakerke, Belgium).

## Results

### Patients

**Fig. 1:**
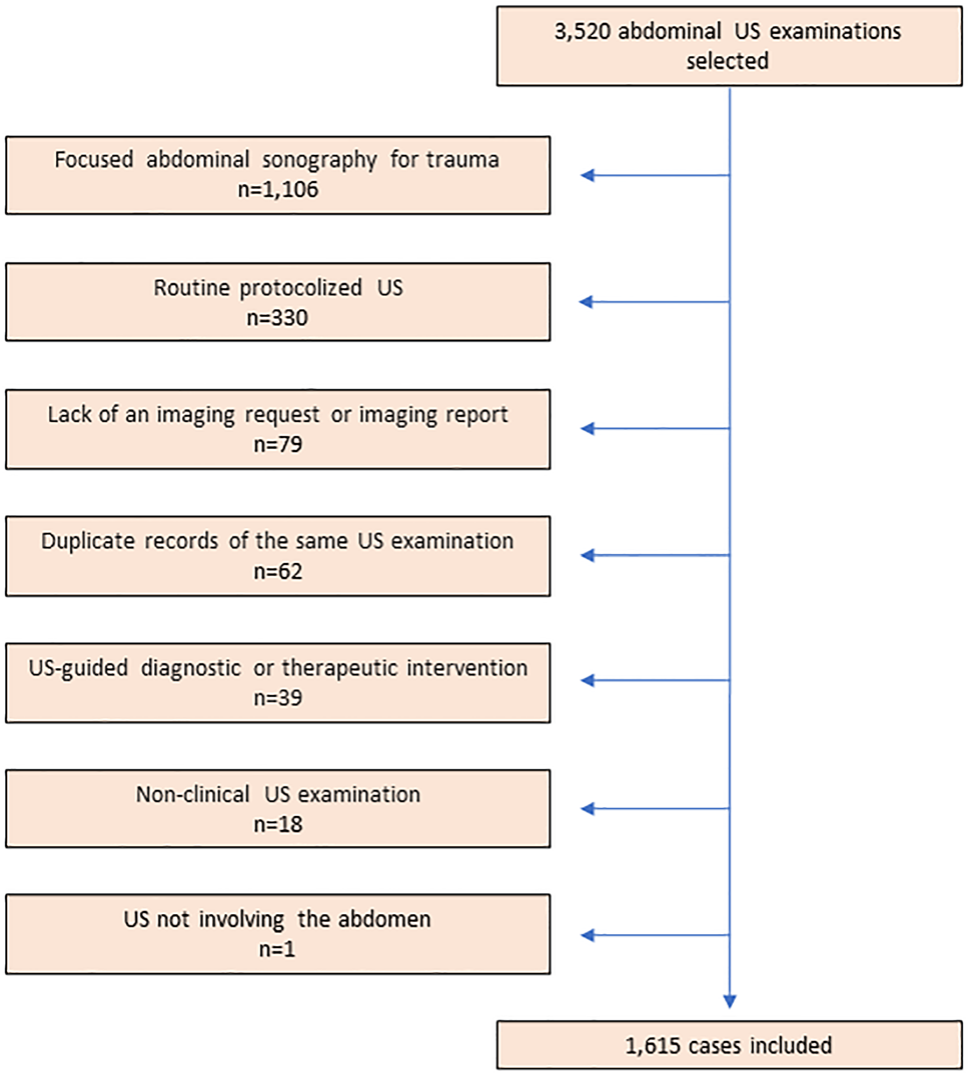
Flowchart of case inclusion

Of 3250 potentially eligible US examinations, 1635 were excluded (Fig. 1). A total of 1615 US examinations remained for final inclusion. Patient gender was male in 893 (55.3%) and female in 722 (44.7%) of these US examinations, and mean patient age ± SD was 44.8 ± 24.3 years (range 0–93 years). Top three requesting departments were surgery (*n* = 709; 43.9%), internal medicine (*n* = 391; 24.2%), and pediatrics (*n* = 107; 6.6%). Top three indications for US were mixed (*n* = 396; 24.5%), gallbladder and biliary ducts (*n* = 274; 17.0%), and other (*n* = 233; 14.4%). Other patient and US characteristics are displayed in supplementary Table 1.

### Negative US examinations

**Fig. 2:**
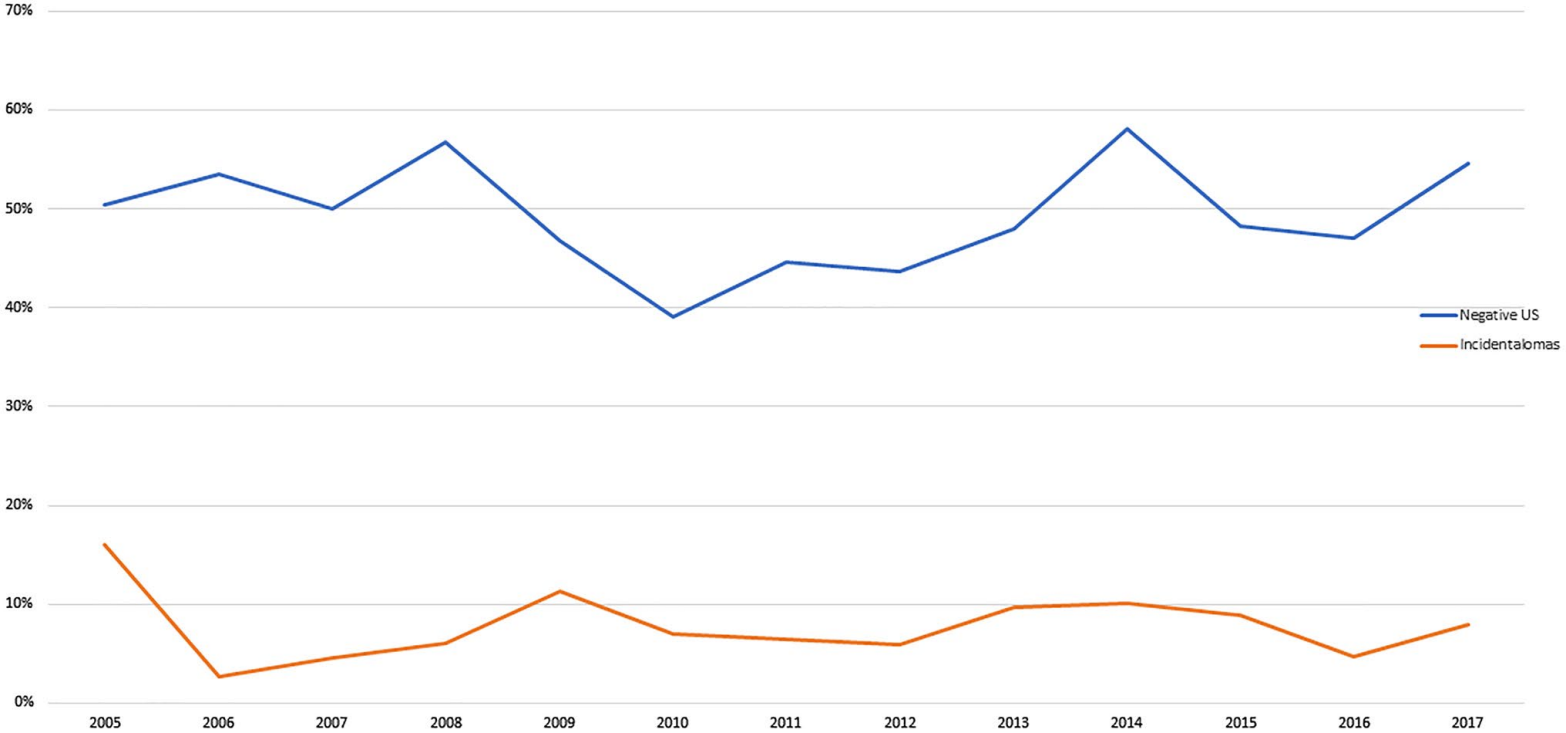
Proportions of US examinations with negative fndings and with an incidentaloma between 2005 and 2017

The total proportion of negative US examinations was 49.2% (795/1615). Total proportions of positive and indeterminate US examinations were 32.6% (526/1615) and 18.2% (294/1615), respectively. There was no significant change in the proportion of negative US examinations over the years (Mann–Kendall tau =  − 0.0256, *P* = 0.951) (Fig. 2). On univariate logistic regression, patient age, requesting specialty, and indication for US were significantly associated with a negative US examination. On multivariate logistic regression, only requesting specialty and indication for US remained significantly associated with a negative US examination (Table 1). Specifically, the likelihood of a negative US examination was significantly higher when requested by anesthesiology [odds ratio (OR) 2.609, *P* = 0.011; note that 35/43 of these US requests were made by anesthesiologist-intensivists working in the intensive care unit, and 8/43 of these US requests were made by anesthesiologists in the emergency department], or when the indication for US was focused on gallbladder and biliary ducts (OR 1.556, *P* = 0.007), transplant (OR 2.371, *P* = 0.005), trauma (OR 3.274, *P* < 0.001), or urolithiasis/postrenal obstruction (OR 3.366, *P* < 0.001). In contrast, US examinations were significantly less likely to be negative when requested by urology (OR 0.423, *P* = 0.014), or when the indication for US was focused on acute oncology (OR 0.207, *P* = 0.045) or appendicitis (OR 0.260, *P* < 0.001) (Table 1).

**Table 1:** Logistic regression on the association between clinical variables and a negative US examination

Variable	Univariate analysis			Multivariate analysis		
	Odds ratio	95% CI	*P*-value	Odds ratio	95% CI	*P*-value
Patient age	1.009^a^	1.005–1.013	** < 0.001**	1.002	0.997–1.008	0.442
Patient gender	1.051	0.863–1.279	0.644	–	–	–
Requesting specialty						
Anesthesiology	3.844	1.907–7.748	**0.002**	2.609	1.248–5.454	**0.011**
Cardiology	1.555	0.801–3.018	0.193	1.087	0.540–2.185	0.816
Cardiothoracic surgery	1.762	0.605–5.131	0.299	1.229	0.408–3.691	0.714
Gastroenterology	1.022	0.662–1.578	0.921	0.756	0.478–1.195	0.231
Internal medicine	1.710	1.333–2.194	** < 0.001**	1.197	0.901–1.589	0.215
Neurology	3.964	1.266–12.411	**0.018**	2.135	0.655–6.957	0.208
Obstetrics and gynecology	1.101	0.546–2.220	0.788	1.080	0.507–2.300	0.841
Pediatrics	1.372	0.912–2.065	0.129	1.183	0.728–1.922	0.497
Pulmonology	2.162	1.006–4.645	**0.048**	1.419	0.643–3.134	0.387
Urology	0.561	0.295–1.066	0.078	0.423	0.214–0.838	**0.014**
Unknown	1.505	0.939–2.412	0.090	1.180	0.715–1.947	0.517
Indication for US						
Abdominal aorta aneurysm	1.213	0.740–1.989	0.444	1.059	0.630–1.782	0.828
Acute bowel pathology	0.941	0.5780–1.533	0.808	1.025	0.615–1.707	0.925
Acute oncology	0.340	0.093–1.237	0.102	0.207	0.044–0.965	**0.045**
Appendicitis^b^	0.244	0.155–0.383	** < 0.001**	0.260	0.162–0.417	** < 0.001**
Gallbladder and biliary ducts^c^	1.588	1.164–2.168	**0.004**	1.556	1.130–2.142	**0.007**
Infection/inflammation	0.831	0.502–1.373	0.469	0.823	0.493–1.373	0.455
Other	1.182	0.853–1.639	0.314	1.171	0.834–1.645	0.363
Transplant^c^	2.699	1.4920–4.883	**0.001**	2.371	1.294–4.342	**0.005**
Trauma^d^	3.239	1.864–5.629	** < 0.001**	3.274	1.855–5.778	** < 0.001**
Urolithiasis/postrenal obstruction	3.676	2.449–5.519	** < 0.001**	3.366	2.220–5.104	** < 0.001**

*CI* confidence interval

^a^Per year of age increase

^b^28, 53, and 90 US examinations were negative, positive, and indeterminate for appendicitis, respectively

^c^Uncomplicated cholecystolithiasis was not regarded as a positive US examination, unless the referring clinician specifically asked for gallstones

^d^Routine protocolized US excluded

^e^Routine Focused Assessment with Sonography for Trauma excluded

### Incidental findings on US

The total proportion of US examinations with an incidentaloma was 8.0% (130/1615). Nine US examinations revealed more than one incidentaloma. Top-three locations of incidentalomas were the liver (*n* = 85; 61.2%), kidneys (*n* = 13; 9.4%), and spleen (*n* = 10; 7.2%) (Table 2). There was no significant change in the proportion of US examinations with an incidentaloma over the years (Mann–Kendall tau = 0.077, *P* = 0.760) (Fig. 2). On both univariate and multivariate logistic regression, patient age and organ(s) evaluated with US were significantly associated with an incidentaloma on US (Table 3). Specifically, the likelihood of an incidentaloma on US was significantly higher in older patients (OR 1.020 per year of age increase, *P* < 0.001) or when the liver was evaluated with US (OR 3.522, *P* < 0.001) (Table 3).

**Table 2:** Incidentalomas per location

Location (or finding)	No	Percentage (%)
Liver	85	61.2
Kidneys	13	9.4
Spleen	10	7.2
Gallbladder and/or biliary ducts	8	5.8
Ascites	6	4.3
Urinary bladder	6	4.3
Upper abdomen n.o.s	2	1.4
Pelvis n.o.s	2	1.4
Pleural effusion	2	1.4
Uterus and/or ovaries	2	1.4
Aorta	1	0.7
Bowel	1	0.7
Testicles	1	0.7

*n.o.s.* not otherwise specifiable

**Table 3:** Logistic regression on the association between clinical variables and an US examination with an incidentaloma

Variable	Univariate analysis			Multivariate analysis		
	Odds ratio	95% CI	*P*-value	Odds ratio	95% CI	*P*-value
Patient age	1.019^a^	1.011–1.028	** < 0.001**	1.020^a^	1.010–1.029	** < 0.001**
Patient gender	0.997	0.695–1.438	0.998	–	–	–
Organ(s) evaluated with US						
Aorta	1.821	1.152–2.879	**0.015**	1.160	0.702–1.917	0.562
Bowel	0.590	0.364–0.956	**0.024**	0.775	0.457–1.314	0.344
Gallbladder and biliary ducts	4.123	2.692–6.316	** < 0.001**	1.484	0.853–2.583	0.162
Kidneys	0.576	0.396–0.836	**0.003**	0.938	0.594–1.479	0.782
Liver	5.873	3.638–9.482	** < 0.001**	3.522	1.843–6.729	** < 0.001**
Pancreas	2.360	1.589–3.507	** < 0.001**	0.918	0.579–1.455	0.716
Scrotum	0.379	0.196–1.568	0.119	–	–	–
Spleen	3.128	2.169–4.510	** < 0.001**	1.572	0.980–2.522	0.061
Urinary bladder	1.827	1.265–2.639	**0.002**	1.495	0.960–2.328	0.075
Uterus and ovaries	1.058	0.477–2.346	0.890	–	–	–

*CI* confidence interval

^a^Per year of age increase

## Discussion

According to clinical experience and anecdotal evidence, the number of imaging examinations that are requested to rule out disease, a practice that is considered to be reflective of defensive medicine, has been and keeps on increasing [15–17]. In an attempt to exercise greater restraint in the use of imaging, a call has been made to establish benchmarks for the acceptable proportion of negative studies [16].

The results of the present study show that nearly half of abdominal US examinations during on-call hours is negative. Monitoring the negative US rate in a radiology practice and providing feedback to clinicians when this proportion exceeds a certain benchmark such as the one that was found in this study, may be a potential tool for radiologists to reinforce their gatekeeper function and reduce overutilization of imaging. Remarkably, the proportion of negative US examinations during on-call hours did not increase between 2005 and 2017, which contradicts our hypothesis. This suggests that US utilization in this setting has not been subject to increased defensive medicine practices in a Western European tertiary health care center. These numbers might be different in institutions or countries with a more defensive healthcare system, such as in the USA. Another interesting finding is that US requests from anesthesiology and certain indications (gallbladder and biliary ducts, transplant, trauma, and urolithiasis/postrenal obstruction) had significantly more negative examinations, while the opposite was true for US requests from urology and certain indications (acute oncology and appendicitis). These findings are probably related to disease prevalence and the clinician’s tendency to either rule out or rule in disease in various clinical settings. For example, it can be expected that patients with acute oncology and genitourinary based pathology would have more positive examinations, because they have a history that predisposes them to complications. Knowledge of which specialties and which indications are more likely to yield a negative or positive examination may be helpful to radiologists in triaging patients for imaging, along with clinical parameters. These variables may vary according to patient demographics and physician’s characteristics.

Incidentalomas, which can be considered an unwanted “byproduct” of imaging, were found in 8.0% of US examinations in the present study, and remained stable over the years. Liver (the largest abdominal parenchymal organ) was by far the most common location with incidental findings, remotely followed by kidneys and spleen. Not surprisingly, liver as organ of US evaluation was also independently associated with a higher risk of finding an incidentaloma on US, along with higher patient age. These data, together with the negative US rate, are important for clinicians and radiologists when deciding on the appropriateness of an US request. They can also be used to inform patients about the expected yield of the US examination and the risk of detecting an incidentaloma (with potential financial and emotional costs), as part of the informed consent and shared decision making process [18].

Benchmarks for the acceptable proportion of negative studies are currently lacking. To the best of our knowledge, only one previous study by Mengou et al. [13] performed a similar investigation. They included a random sample of 1716 acute abdominal CT scans that were made during on-call hours between 2005 and 2019 [13]. They reported a negative scan proportion of 40.0%, which did not show any significant temporal fluctuations during their study time frame [13]. Their negative scan rate for CT (40.0%) was lower than was found for US in the present study (49.2%). This may be explained by the fact that the clinical threshold to request a US examination is lower than that for CT because the former does not use any ionizing radiation and is less expensive. Because of this lower clinical threshold, the disease prevalence and the proportion of patients with pathological findings on US is also lower. Previous research has shown that there is large variability in the prevalence of incidentalomas across different imaging modalities [3]. Of note, the frequency of incidentalomas of 8.0% that was found in the present study almost exactly matches the findings of a study by Mills et al. [19] on incidentalomas in 1000 abdominal US examinations.

This study had some limitations. First, the results of this study apply to The Netherlands, where malpractice litigation has been reported to happen less frequently than in other countries such as the USA [20]. In countries where malpractice suits are more common, the rate of negative imaging examinations may be higher due to more defensive medicine practices. Second, this study was performed in a tertiary care setting. The results may be different in non-tertiary care centers with different patient populations. Third, all US examinations were performed by radiology residents, either without or with supervision from a radiologist. Differences in observer expertise and experience may have affected the results. However, this reflects clinical practice. Fourth, our findings may not be directly applicable to other countries such as the USA where the selection of patients with suspected acute abdominal pathology for imaging, and the ones who perform and interpret these acute imaging examinations, are different than in the Netherlands because of societal and healthcare system differences [10, 11]. Fifth, no reference standard was used to confirm the US findings in this study, because additional cross-sectional and pathologic examinations are often lacking, particularly when US is negative. Sixth, 18.2% of US examinations was indeterminate, but it remained unclear which proportion was due to obesity because this was not consistently prospectively recorded. In conclusion, nearly 50% of abdominal US examinations during on-call hours are negative, and 8% reveals an incidentaloma. Requesting specialty and indication for US affect the likelihood of a negative examination, and higher patient age and liver evaluations increase the chance of detecting an incidentaloma in this setting. These data may potentially be used to improve clinical reasoning and restrain overutilization of imaging.

## Supplementary Information

Below is the link to the electronic supplementary material.

Supplementary file1 (DOCX 15 kb)

## Abbreviations

OR: Odds ratio
FAST: Focused Assessment with Sonography for Trauma
US: Ultrasonography
