# Supplementary material for: On-call abdominal ultrasonography: the rate of negative examinations and incidentalomas in a European tertiary care center
Source: Abdom Radiol (NY). 2022 Apr 29;47(7):2520–6. doi: 10.1007/s00261-022-03525-1 (PMC9226090; doi:10.1007/s00261-022-03525-1)
Supplement: Supplementary file 1 — Supplementary file1 (DOCX 15 kb) [file 261_2022_3525_MOESM1_ESM.docx]

**Supplementary Table 1.** Patient and US characteristics.

| **Variable** | **No.** | **Percentage** |
| --- | --- | --- |
| Patient age (years) | 44.8^a^ | 24.6^b^ |
| Patient gender |  | |
| *-Female* | 722 | 44.7% |
| *-Male* | 893 | 55.3% |
| Requesting specialty |  | |
| *-Anesthesiology* | 43 | 2.7% |
| *-Cardiology* | 37 | 2.3% |
| *-Cardiothoracic surgery* | 14 | 0.9% |
| *-Dermatology* | 1 | 0.1% |
| *-ENT* | 2 | 0.1% |
| *-Gastroenterology* | 95 | 5.9% |
| *-Internal medicine* | 391 | 24.2% |
| *-Neurology* | 16 | 1.0% |
| *-Neurosurgery* | 7 | 0.4% |
| *-Obstetrics and gynecology* | 33 | 2.0% |
| *-Orthopedics* | 4 | 0.2% |
| *-Pediatrics* | 107 | 6.6% |
| *-Plastic surgery* | 1 | 0.1% |
| *-Pulmonology* | 29 | 1.8% |
| *-Rheumatology* | 2 | 0.1% |
| *-Surgery* | 709 | 43.9% |
| *-Urology* | 47 | 2.9% |
| *-Unknown* | 77 | 4.8% |
| Indication for US |  | |
| *-Abdominal aorta aneurysm* | 75 | 4.6% |
| *-Acute bowel pathology* | 79 | 4.9% |
| *-Acute liver failure* | 4 | 0.2% |
| *-Acute oncology* | 15 | 0.9% |
| *-Appendicitis* | 171 | 10.6% |
| *-Gallbladder and biliary ducts* | 274 | 17.0% |
| *-Infection/inflammation* | 77 | 4.8% |
| *-Mixed* | 396 | 24.5% |
| *-Other* | 233 | 14.4% |
| *-Transplant*^c^ | 57 | 3.5% |
| *-Trauma*^d^ | 72 | 4.5% |
| *-Urolithiasis/postrenal obstruction* | 162 | 10.0% |

Notes:

^a^ Mean

^b^ Standard deviation

^c^ Routine protocolized US excluded

^d^ Routine Focused Assessment with Sonography for Trauma excluded
